# Stress responses of plants through transcriptome plasticity by mRNA alternative polyadenylation

**DOI:** 10.1186/s43897-023-00066-z

**Published:** 2023-09-28

**Authors:** Jiawen Zhou, Qingshun Quinn Li

**Affiliations:** 1 Key Laboratory of the Ministry of Education for Coastal and Wetland Ecosystem, College of the Environment and Ecology, Xiamen University, Xiamen, 361102 Fujian China; 2 Biomedical Sciences, College of Dental Medicine, Western University of Health Sciences, Pomona, CA 91766 USA

**Keywords:** Alternative polyadenylation, RNA processing, Transcriptome diversity, Biotic and abiotic stress, Stress response

## Abstract

The sessile nature of plants confines their responsiveness to changing environmental conditions. Gene expression regulation becomes a paramount mechanism for plants to adjust their physiological and morphological behaviors. Alternative polyadenylation (APA) is known for its capacity to augment transcriptome diversity and plasticity, thereby furnishing an additional set of tools for modulating gene expression. APA has also been demonstrated to exhibit intimate associations with plant stress responses. In this study, we review APA dynamic features and consequences in plants subjected to both biotic and abiotic stresses. These stresses include adverse environmental stresses, and pathogenic attacks, such as cadmium toxicity, high salt, hypoxia, oxidative stress, cold, heat shock, along with bacterial, fungal, and viral infections. We analyzed the overarching research framework employed to elucidate plant APA response and the alignment of polyadenylation site transitions with the modulation of gene expression levels within the ambit of each stress condition. We also proposed a general APA model where transacting factors, including poly(A) factors, epigenetic regulators, RNA m^6^A modification factors, and phase separation proteins, assume pivotal roles in APA related transcriptome plasticity during stress response in plants.

**Graphical Abstract:**

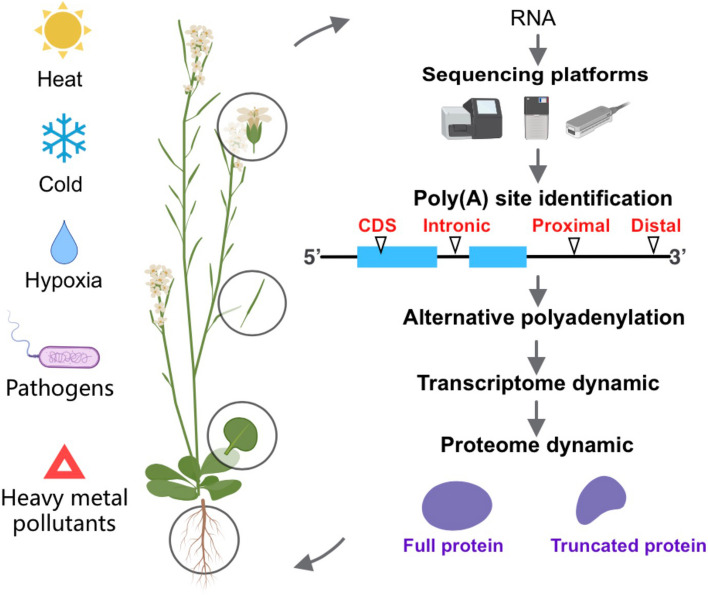

## Introduction

Messenger RNA polyadenylation is a pre-mRNA 3’ end processing mechanism in co-transcriptional regulation during gene expression. This mechanism determines the end of mRNA 3’ UTR (untranslated region) by cleavage on the pre-mRNA and adding a polyadenine tail (Mitschka and Mayr 2022). Alternative polyadenylation (APA), a phenomenon where transcripts of the same gene use more than one poly(A) site, is very pervasive in plants. About 70% of *Arabidopsis* genes and 60% of rice and sorghum genes possess at least two poly(A) sites (Wu et al. 2011b; Fu et al. 2016). As a mechanism that maximizing transcriptome diversity and contributes to gene expression complexity of higher eukaryotes, APA dynamic is of great importance in both plants and animals (Xing and Li 2011; Tian and Manley 2017).

Dysregulated 3ʹ end processing, including the gain or loss of APA and global changes in poly(A) site usages, is involved in various of human diseases including immunity, cancers, blood pathology and nerve system degenerations (Gruber and Zavolan 2019). For instance, the poly(A) signal alters from AAUAAA to AAUGAA in *FOXP3* leads to an immune dysregulation syndrome (Bennett et al. 2001); widespread poly(A) site switch to intronic loci inactivates tumor suppressor genes in leukemia (Lee et al. 2018) and extensive APA were found associated with leukemia at the single cell level (Ye et al. 2019a). In plants, APA events are highly involved in development, environmental responses and disease resistance. Examples include APA in genes such as *FCA*, *FPA*, *FLC* and *HLP*, which regulate flowering time, as well as in *DOG1* for seed germination, *IBM1* for DNA methylation, and *Nrt1.1* along with *FIP1* for nitrate signaling (Saze et al. 2008; Xing and Li 2011; Cyrek et al. 2016; Bernardes and Menossi 2020). The *Arabidopsis* gene *CPSF30,* encoding a poly(A) factor that participates in the generation of APA dynamics for many development and stress response pathways, is an APA gene itself (Delaney et al. 2006; Thomas et al. 2012; Liu et al. 2014). Recently, the APA dynamics of the rice *XRN1* encoding an exonuclease homologue has been found to play a role in pollen development (You et al. 2021).

Systematic searches of APA through various of transcriptome wide 3’-end sequencing methods were carried out in many species from diatoms to rice (Wu et al. 2011a; Wu et al. 2012; Fu et al. 2019; Wu et al. 2019; Mo et al. 2021), and even at the single-cell type or single-cell levels in *Arabidopsis* (Cao et al. 2019; Long et al. 2021). Compilation of these experiments are a transcriptome-wide collection of APA sites throughtout different genomes, and offer a rich resource for gene-specific investigations. Furthermore, epigenomic modifications have also been associated to APA regulation although there are key links awaiting elucidation (Lin and Li 2022).

In this review, we focus on the APA dynamic features and how APA affects, or be affected, when responding to biotic and abiotic stresses in plants, horticulture crops included.

## APA dynamics and regulation

### Dynamic features

Messenger RNA polyadenylation is such a changing and dynamic mechanism that closely accompanies an organism’s growth, development, and adaptation. It exhibits variability in the selection of different poly(A) sites, the frequency of poly(A) site usage, and the length or composition of poly(A) tails.

*Poly(A) sites*. APA genes typically possess multiple poly(A) sites, primarily located in the 3’ UTR, while others are found in the 5’UTR, introns, intergenic regions, or exons (Guo et al. 2016). The existence of the alternative poly(A) sites leads to different transcripts produced from the same gene, forming the basis of APA regulation in gene function. Poly(A) site usage (frequency) and switching (in comparison of two sites) among alternative sites are the most fundamental and complex feature in all kinds of APA regulations leading to different fate of the APA variants thus regulating gene expression (Wu et al. 2011b). If an APA site in the CDS or intronic regions is used, it would generate shortened mRNA and/or a truncated protein. Additionally, the mRNA may also be degraded due to lack of a stop codon or non-sense mediated decay, triggering a negative regulation (de Lorenzo et al. 2017). If an APA site is at 3’UTR, the resulting transcript may be shortened or lengthened, to exclude or include regulatory cis-elements, respectively, subjected to additional regulation of the gene (Xing and Li 2011; Tian and Manley 2017). Finally, if a prolong APA site is used, it could extend into and interfere with the expression of the next gene (Guo et al. 2016). Thus, selecting a poly(A) site is substantial in regulating gene expression.

*Poly(A) tails*. It generally protects mature mRNA from random degradations. Poly(A) tail length is another dynamic feature that varies across species, gene types, tissues and development stages (Jalkanen et al. 2014). However, recent reports indicated that poly(A) tails are not solely composed of adenine (A). In *Arabidopsis*, approximately 10% of poly(A) tails contain guanosine (G), ranging from 0.8% to 28%, and studies have shown that the presence of G in poly(A) tails enhance translational efficiency (Zhao et al. 2019a). Similar results were also presented (Liu et al. 2019).

These dynamic characteristics of polyadenylation contribute to the intricate regulation of gene expression and its functional outcomes.

## Factors defining APA sites

APA are mainly regulated by the polyadenylation factor complex (PA complex) and the signal cis-elements located upstream or near the poly(A) site in plants. These two parts work together to choose a poly(A) site, complete cleavage, and polyadenylation with a number of related proteins and/or enzymes (Tian and Manley 2017).

*Poly(A) signals*. The hexamer “AAUAAA” is the most common poly(A) signal in mammal, followed by “AUUAAA”, “UAUAAA”, “AAAAAA” and “AGUAAA”; therefor, poly(A) sites could be predicted in some extent by scanning these poly(A) signals (Slutskin et al. 2019). In red alga and diatoms, “UAAA” tetramer as well as “UAA” triplet are dominant poly(A) signals, respectively, and their frequency in the near upstream elements (NUE; 30–50 nts upstream of a poly(A) site) could be around 80% (Zhao et al. 2019b). However, in higher plants, the dominant poly(A) signal “AAUAAA” only occupies about 10% in the NUE region, and its one-nucleotide variants also only reach 20–30% in most cases (Loke et al. 2005; Lin and Li 2022). These observations leave open a wide speculation on what plays significant role in determining poly(A) site selection in plants. On the other hand, there are Far-Upstream Elements (FUE, > 50 nts upstream of a poly(A) site) in plants that are required in regulating poly(A) site selections, since the signals are widely detectible on almost all genes, in contrast to animals where only some genes possess such a upstream element (Ye et al. 2021).

Polyadenylation related cis-elements are also targets of RNA-binding proteins, the interaction of them determines the poly(A) site selection of pre-mRNA in eukaryotic cells. There are evidence that plant 3’ regulatory regions have the potential to increase gene expression level in some target genes (Bernardes and Menossi 2020). UA-rich elements tend to have strong effects on gene expression and their mutations caused mRNAs possessing aberrantly long 3’UTRs (Shalem et al. 2015). A computational model in yeast set up to show the correlation between the strength of these elements in 3’ ending and their expression variability (Shalem et al. 2015).

*Polyadenylation complex*. The trans-acting proteins involved in the PA complex is highly conserved in mammals and yeast. The human PA complex including cleavage and polyadenylation specificity factor (CPSF), cleavage stimulation factor (CstF), cleavage factor I (CFIm) and cleavage factor II (CFIIm), poly(A) polymerase and some other proteins, with homologs present in yeast (Yoon and Shi 2022). By comparison, most of these poly(A) factors were identified in plants (Hunt et al. 2008; Thomas et al. 2012), and many of them were confirmed by functional analyses (Lin and Li 2022). The plant PA complex component CPSF30 and FY are the most studied factors. CPSF30 and FY bind to the “AAUAAA” or NUE signal, and the CPSF30 YTH domain bind to N6-methyladenosine (m6A)-modified UGUA signals as reviewed recently (Lin and Li 2022). However, further understanding of the functions of these PA proteins in APA is necessary to elucidate their roles in stress responses.

A recent investigation has shown that RNA structure may also contribute to successful cleavage of the pre-mRNA where an RNA structure containing two close-by single-stranded regions that is associated with some polyadenylation events (Liu et al. 2021). This work indicated that maintaining a single-strand status of the main poly(A) signal (e.g., AAUAAA or NUE) is important for polyadenylation in the nucleus. However, the involvement of such a structural feature in APA or selection of a poly(A) site remains to be demonstrated.

## APA dynamic in response to abiotic stress

The abiotic stresses we focus on herein are due to environment pollution, climate change and special habitats that plants face, including heavy metal cadmium (Cd), heat shock, cold, high salt and hypoxia. For biotic stresses, we will have several common diseases in industrial crops and fruits caused by pathogenic microorganisms, including bacteria, fungi and viruses.

### Cd

Environment pollution caused by heavy metals (like mercury, cadmium, lead and arsenic) poison and limit the growth of both plants as well as animals. In mammalian cells, arsenic stress induces the use of proximal poly(A) sites and leads to the shortening of 3'UTRs, particularly in genes related to "differentiation and proliferation", as a means to preserve RNA transcripts and prevent decay (Zheng et al. 2018). Plants also exhibit some adaptive mechanisms in exposed to Cd stress. Cd is not considered an essential element for plants. However, due to its widespread presence as a strong toxic heavy metal in water and soil, plants can absorb and accumulate it through their roots. This accumulation can subsequently lead to iron deficiency and detrimental effects on photosynthesis in plants (Siedlecka and Baszyńaski 2010). Cd stress stimulates AS (alternative splicing) and the expression of a variety of transacting factors (TFs), including zinc finger TFs, AP2-EREBP TFs, WRKY TFs, NAC TFs and MYB factors (He et al. 2015). The long transcript of the gene encoding plant poly(A) factor CPSF30-L was found to be enriched under Cd treatment, indicating its potential involvement in Cd stress response (Song et al. 2021).

As an organ directly in contact with soil, root is the first tissue to be affected. Cd stress causes an impact on root growth and morphology in *Arabidopsis*, resulting in altered polyadenylation clusters (PACs) in genes associated with Cd ion response (*GSH1, GDH1*) and osmotic stress (*ERD, ERD6, ERD14*) (Cao et al. 2019). Conversely, downregulated PACs are enriched in genes related to root hair development (*ACT, CSLD, MRH, RHS, TUB2, XTU*), translation initiation (*elF3),* and non-coding RNA (ncRNA) processing (*AGO)*; significant poly(A) sites switching has also been observed in genes such as *RH18, SKIP, RAP2.6, SCL8* and *UPBEAT1* (Cao et al. 2019). Notably, *GSH1, GDH1* and *ERD* gene family were confirmed for their role in Cd tolerance: *GSH1* is a *yAP-1* related metal and drug resistance gene while *GDH1* is redox reaction related, which encodes NADP-dependent glutamate dehydrogenase (Moye et al. 1985; Wu and Moye-Rowley 1994); the *ERD* gene family is induced by stress, particularly in cold and dehydration (Shinozaki 2000).

In rice, different concentrations of Cd activated distinct pathways, where low Cd concentrations primarily affected DNA repair pathways and high Cd concentrations influenced cell wall formation pathways (Ye et al. 2019b). DNA repair related genes *OsRPA1a* and *OsEXO1* used proximal poly(A) sites in low Cd condition, indicative the potential influence of APA on these gene expression (Ye et al. 2019b).

These case studies emphasize the importance of APA in response to Cd toxicity. APA increases mRNA transcripts dynamic and further modulates the level of the involved proteins in remediating Cd stress.

### Salt

Plants grown in saline soils face the sinister living conditions. Researches indicate that stress sensing and signaling components play a vital role in the regulation of plant salinity stress responses. Several genes and factors (TF, NHX and SOS) were identified to participate in this process (Deinlein et al. 2014). APA was also found to participate in the response to high salt. In *Spartina alterniflora*, an invasive monocot species, exposure to high salt stress induced 3’ UTR lengthening in ion transporter transcripts by favoring the utilization of distal poly(A) sites (Wang et al. 2022). Concurrently, the expression level of poly(A) factors were altered under high salt stress, with the upregulation of *SaCFIm25* and *SaCFIm68*, along with the downregulation of *SaCPSF100* and *SaCPSF30* (Wang et al. 2022). It is worth noting that *CFIm25* and *CFIm68* are two factors promoting the usage of distal poly(A) sites in mammalian cells (Masamha et al. 2014). This phenomenon of 3' UTR lengthening may contribute to the expression of proteins involved in salinity tolerance through the involvement of transposable elements (TEs). Unlike arsenic response in mammalian cells, *S. alterniflora* 3’ UTR lengthening did not impact global mRNA accumulation, but it influenced mRNA stability and protein level in certain instances (Wang et al. 2022).

Compared to salt tolerant plants, salt sensitive plants may exhibit stronger variation in APA profile. Under salt stress, *Arabidopsis thaliana* (salt-sensitive) and *Eutrema salsugineum* (salt-tolerant) both tend to utilize distal poly(A) sites, but *Arabidopsis* presents more significant changes in gene expression via APA compared to *Eutrema* (Ma et al. 2022). In *Arabidopsis*, APA genes were enriched in “response to salt stress” category, but not the same in *Eutrema* where salt tolerance-related TFs (*CIPK21* and *LEA4-5*) showed differential APA patterns (Ma et al. 2022). This suggests that adapted species could orderly cope with salt stress with mild APA dynamic profile compared to non-adapted specie. This in some extend confirms APA dynamic to be a strategy when plants response to stress. In *Arabidopsis*, poly(A) factor *FY* and *AtCPSF30* affected APA of two transcription regulators, *AT3G47610* and *AKR2*; and further studies demonstrated the APA of two genes were associated with increased rates of green cotyledon retention and seed germination under salt stress (NaCl) compared to control plants (Yu et al. 2019). Thus, plant poly(A) factors have the potential responding to salt stress via altering the APA of transcription regulators and others.

### Hypoxia and oxidative responses

Another challenge to plants undergoing extreme weather conditions (rainstorm, flooding and drought) is hypoxia and oxidation stresses. While the majority of poly(A) sites are situated within the 3' UTRs, a small percentage are found in non-3' UTR regions, indicating the potential for regulation through switching to non-3' UTR poly(A) sites (Guo et al. 2016). Hypoxia leads to variable poly(A) site usages in *Arabidopsis*, and this mainly reflected in the dynamic of noncanonical poly(A) sites (de Lorenzo et al. 2017). Upregulated isoforms in CDS, 5’ UTRs, introns were 6%, 13%, 10%, respectively, compared to just 0.3% of isoforms within 3’ UTR were upregulated in hypoxia (de Lorenzo et al. 2017). Noncanonical APA often leads to unstable isoforms and produces no protein product. There might be some yet unknown factors to regulate other genes when responding to hypoxia.

Direct relationship of APA and oxidative stress came from the analysis of *Arabidopsis* mutant *oxt6,* which is caused by the disruption of *CPSF30* via a T-DNA insertion, found by a genetic screening of oxidative tolerance (hence *oxt*) (Zhang et al. 2008). The transcriptional profiles of *oxt6* revealed modifications in genes associated with reactive oxygen species (ROS), and noteworthy alterations in poly(A) sites were identified, although poly(A) tail length appeared unaffected (Zhang et al. 2008). Specifically, genes related to glutaredoxins and thioredoxins exhibited varying expression levels that correlated with ROS tolerance (Zhang et al. 2008). A broad range APA sites were changed in the *oxt6* mutant, in which calmodulins, by extension calcium signals, were indicated to be involved in the function of CPSF30 (Liu et al. 2014). Indeed, APA site choices were altered significantly in the *oxt6* mutant for gene *LHCB4.1* (encoding light harvesting complex II subunit), and this alteration was linked to responses under light and temperature stresses (Chen 2011). In the context of rice, APA is implicated in drought-responsive genes, contributing to drought tolerance by facilitating the accumulation of osmoprotectants and scavenging of ROS (Cui et al. 2015; Ye et al. 2019b).

These findings highlight the involvement of APA in ROS management in plants. Since ROS are common on numerous stress responses beyond hypoxia and oxidative stresses, e.g., heat and cold, flooding and drought, and heavy metal etc., it is expected that APA could be substantially involved in many other pathways.

### Heat and cold

Global warming is not just reflected on the raising temperature, but extreme weathers like excessive heat or cold bring more challenges to plants. Thus, adaptation of plants to these extremes could be consequential for their survival.

*Heat stress*. Heat shock protein coding genes *OsHSP82A*, *OsHSP40*, *OsHSP74.*8, *HSP101* and heat shock transcription factor *OsHsfB2c* were shown to be dynamic in APA after heat shock in rice; they also show at high expression levels (Wilkins et al. 2016; Ye et al. 2019b). In *Arabidopsis*, *CPSF30* mutant *oxt6* also exhibited a set of genes were strongly induced by heat shock, and the expression of these genes were changed in an *AtCPSF30*-dependent manner (Zhang et al. 2008).

Poly(A) tail length (PAL) has also been found to be responsive to heat shock in plants. PAL is proven to be interrelated with protein expression level (Lima et al. 2017) and plays a vital role in transcripts’ biological functions (Jalkanen et al. 2014). Analysis using APAL-seq (Assay for PAL-seq) by PacBio sequencing platform has revealed the PAL dynamic in transcription level in *Arabidopsis* in response to heat shock: heat shock protein coding gene *HSP70* and other HSP-related genes, exhibit significant PAL changes, and heat shock pattern (abrupt heat stress and gradual heat stress) affected their response (Wu et al. 2020). In this study, it was also confirmed that poly(A) tail elongation influences the expression level of the HSP70 protein (Wu et al. 2020).

*Cold stress*. *Japonica* and *indica* are two common subspecies of cultivated rice (*Oryza sativa*), and they diverge substantially in cold tolerance where *japonica* is a typical high-altitude-growing rice and exhibit low-temperature tolerance via a ROS-dominated adaptation mechanism (Zhang et al. 2016). A study based on poly(A) tag sequencing (PAT-seq) found that *japonica*-specific PACs (like *GSS* and *GR3*) were overrepresented in glutathione (GSH) metabolism pathways, leading to a high GSH content (Zhou et al. 2019). GSH concentration increases with many kinds of stresses, cold included, which is ascribed to a raising capacity of antioxidative protection (Ye et al. 2019b). Thus, APA is a potential factor in cold response via GSH-ROS related tolerant mechanism.

Cold response in rice shows a tissue-specific character. An obvious poly(A) site switching to introns were found only in young rice leaves; further domain analysis in these genes indicated short isoforms produced by APA had fewer functional domains, which suggests a potential means by which APA turned off these gene expression under stress in specific tissues (Shen et al. 2011).

Also in rice, polyadenylation of long non-coding RNAs (lncRNAs) are involved in response to heat as well as cold stresses. It was concluded that lncRNAs with a poly(A) tail were downregulated significantly, and many downregulated polyadenylated lncRNAs were co-expressed with stress-related genes (Yuan et al. 2018). While no specific APA data were shown, these results indicated potential roles of polyadenylation in heat and cold stress response by lncRNAs.

In *Arabidopsis*, a novel iso-seq based transcriptome study found that cold induced gene *AT1G11280* and *AT3G13110* switched to use proximal poly(A) sites while gene *AT5G5320* switched to use a distal poly(A) site, and they all increased expression level and had a dramatically expression peak in the night (dark) time compared to the day (light) time (Zhang et al. 2022). It would be interesting to explore further if such an association has a causal effect to cold stress regulation.

### Circadian rhythms

Circadian rhythms could be reckoned as a long term, complex and regular response to integrated environmental factors mainly include temperature and light/dark cycles (Gregson et al. 2005). Almost all organisms have its own circadian rhythms. Alternative splicing (AS) and APA factors also exhibit circadian and diurnal rhythmicity (Yang et al. 2020). APA response to this long-term light/dark switch, and RNA transcripts undergo rhythm-induced APA both in animals as well as plants (Liu et al. 2013; Wang and Ma 2013).

The oscillation of APA influenced mRNA stability, protein level and finally circadian functions in mammal lines: mouse cold-induced proteins Cirbp and Rbm3 caused amplitude of core circadian gene expression by controlling poly(A) site oscillating (Liu et al. 2013). A genome-wide analysis in mouse liver also unveiled that 57.4% of expressed genes underwent APA and 2.9% of genes switched their poly(A) sites on the basis of circadian rhythm (Gendreau et al. 2018).

In *Arabidopsis*, three feedback loops (core, morning and evening loop) comprise the clock circadian basis (Wang and Ma 2013). The three loops are regulated in several mechanisms including at the transcriptional and post-transcriptional levels via regulating APA and 3’ UTR profiles (Mateos et al. 2018). Further investigations conducted in *Arabidopsis* underscore genome-wide regulation of APA response to circadian clock and rhythmic light exposure: PAT-Seq data revealed that APA transcripts could be classified into rhythmic and arrhythmic groups according to their diurnal rhythm features (Lin et al. 2021b). Histone markers activation might contribute to the differentiation of the two groups, and these groups also displayed distinct poly(A) signals, showing uncommon A/C and U/G usages in potential cis-elements (Lin et al. 2021b).

A mathematical model was created to describe the dynamics of APA transcripts associated with rhythmic expression: a molecular-diode mechanism was calculated, which maintains dynamically balanced production rate of total protein via regulating APA induced transcript oscillation (Ptitsyna et al. 2017). The connection between plant polyadenylation and circadian control was also demonstrated by the mutation of a poly(A) factor, PCFS4, which resulted in altered expression of key circadian clock protein encoding genes TOC1 and CCA1 (Xing et al. 2013).

Thus, APA responses to dark/light to influence the transcription rhythm and plays an important role in clock circadian feedback loops.

## APA dynamics in response to biotic stresses

Besides environmental factors, plants also face the invasion by other living beings including bacteria, fungi and viruses. Phytopathogenic microorganisms have a significant effect on the survival, development and productivity of plants. Disease resistance genes (R genes) encode immune receptor proteins, which are vital for plant against pathogenic microorganisms. Methylation and retrotransposon domestication mediated APA are involved in R genes related pathways. R gene *RPP7* is regulated by the histone mark H3Kme2-mediated APA through a TE inserted in its intron, and this model also slightly affected *RPP7* expression level (Tsuchiya and Eulgem 2013). Enhanced Downy Mildew 2 (EDM2) is assumed in tuning this *RPP7* regulation model as a genome CHG methylation regulator via affecting APA of *IBM1* (Lei et al. 2014). The epigenetic marks on the TE of RPP7 also served as a model revealing the connection between epigenetic and APA (Ma et al. 2014a).

CPSF30 is another protein involved in the damage recovery after plant affected by pathogens. Plant CPSF30 is required for lesion formation in gene *mips1* via salicylic acid dependent signaling, and this is an important component of plant immune responses (Bruggeman et al. 2014).

### Bacteria

Bacterial blight is a common bacterial infection in rice caused by *Xanthomonas oryzae pv. Oryzae (Xoo)*, resulting in wilt seedlings and dark brown necrotic leaf spots with yellow halos (Hollaway et al. 2007). The resistance and symptoms related genes were both found to use APA in Rice (Zhang et al. 2015). Bacterial blight resistance genes (*OsRti1a, OsDR8, OsWAK14, R family OsSSI2*, etc.), stress related genes (*SRZ1, OsPP18, OsDREB6, OsHSP71.1*, etc.) and chlorophyll biosynthesis genes (SGR, YL1, ASL2, OsFBK12, etc.) are all have an apparent 3’UTR lengthening (most) or shortening (Ye et al. 2019b) under *XOO* attack. However, the change of APA profiles awaits confirmation at the gene expression level.

In *Arabidopsis*, CPSF30 activity is necessary for resistance to *Pseudomonas syringae*, and the *oxt6* mutation also suppresses cell death in other lesion-mimic mutants, such as *mitogen-activated protein kinase4* and *lesion-simulating disease1* (Bruggeman et al. 2014). These imply that CPSF30 play a role in cell death in response to phytopathogen damage. It would be interesting to examine if there are transcriptome level APA in assisting such immune responses.

### Fungi

Rice blast, sheath rot and false smut are the top three economically vital diseases in rice (Khanale et al. 2022). Rice blast is caused by an ascomycetes fungal plant pathogen *Magnaporthe oryzae*, which leads to a yield loss of 60%-100% by damaging most plant parts at all living stages (Eaty et al. 2020). A protein elicitor isolated from *M. oryzae* MoHrip1 could induce rice blast-resistance, and the RNA-seq data of MoHrip1 induced differentially expression genes (DEGs) were found to be associated with several pathways (Shun et al. 2016). Among them, rice blast induced jasmonate biosynthesis pathway is the most likely influenced by APA dynamics. Two jasmonate biosynthesis-related genes were found to have APA dynamic: jasmonate ZIM-domain gene *OsJAZ1* was found to use its proximal poly(A) site with increased expression level, while jasmonate biosynthesis catalyzer *OsAOC* preferred to use the distal APA site with decreased expression after MoHrip1 treatment (Ye et al. 2019b).

### Viruses

Rice strip is a viral disease that harms the rice production, especially in the *japonica* varieties. Rice strip virus (RSV) is transmitted by the small brown planthopper (SBPH) (Sun and Jiang 2006). Analysis of de novo transcriptome assembly data through sequencing two rice cultivars (Liaonong 979 and Fengjin, resistant and susceptible to RSV, respectively) before and after RSV infection found that many DEGs were induced by RSV (Zheng et al. 2013). A subsequent study on APA using the same data found that APA contributed to the resistance of Liaonong 979. In Liaonong 979, the gene *OsNPR1* switched to use a distal poly(A) site and displayed reduced expression, thereby decreasing the rice attraction by SBPH; gene *OsWRKY46, OsRLCK07* and *OsRAR1* switched to use proximal poly(A) sites and displayed increased expression, also resulting the decreased rice attraction to SBPH, enhancing insect resistance; but these are not found in susceptible Fengjin (Ye et al. 2019b).

Similar to the infection caused by *XOO*, genes involved in porphyrin and chlorophyll metabolism pathways, such as *YGL1* and *NYC1,* underwent changes in both3’ UTR profile as well as expression level after RSV infection, and this alteration was reasoned to resulted in yellow leaves and leaf diseases (Ye et al. 2019b). While these conclusions were drawn from data analysis, they provide valuable guidance for subsequent experimental confirmations.

## Features of stress induced APA genes

Across these various studies, a consistent pattern emerged showcasing widespread poly(A) site switching and distinct differential expression levels in response to many stress conditions in plants. Tables 1 and 2 provide an overview of these dynamic APA genes and their corresponding APA characteristics, encompassing poly(A) site utilization, alterations in expression levels and changes in poly(A) tail length (only in heat shock study). Notably, a majority of the identified APA genes exhibit a strong correlation with stress responses, while others assume to have roles in stress-related pathways. For instance, in rice blast, genes like *OsJAZ1* and *OsAOC* contribute to jasmonate biosynthesis. Moreover, certain genes linked to phenotype changes, such as *NYC1, YGL1* and *SGR*, which were associated with plant diseases, wherein injury prompts leaf development at an early stage.

**Table 1:** Dynamic features of plant APA genes related to abiotic stress responses

Abiotic stress						
Stress	General trend of 3’UTR	Poly(A) site usage	Gene		Expression level	Reference
Cd	/	proximal	*AT5G54370(long-time stress); OsPrx1, OsRPA1a, OsEXO1*		↑	(Cao et al. 2019; Ye et al. 2019b; Zhou et al. 2019)
			*AtBGLU23, AtTUB2, AtCAP1, AtPGY1, AtCSLE 1, AtRH19*		/	
		distal	*AtBT4, AtAPI2, AtHP60, AtCYP81F 4, AtARP1, AtNOG1- 1, AT5G54370 (short-time stress), AtLOS1, AtASK2, OsPOD, CPSF30*		/	
High salt	lengthening	distal	*AT4G23050, AT5G62470, Thhalv10011676m, Thhalv10014879m*		↑	(Ma et al. 2022; Wang et al. 2022)
Hypoxia	/	non-3’ UTR	*RBOHD, TPS8, GID1A, AT3G46530, AT3G23030, AT5G46780*		↑	(Cui et al. 2015; de Lorenzo et al. 2017; Ye et al. 2019b)
		distal	*NADP-ME2, OsAPX1, OsAPX2*		↑	
			*DCA1, OsRZFP34*		↓	
Heat	lengthening	proximal	*OsHSP74.8, OsHSP40, HSP101,*		↑	(Wilkins et al. 2016; Ye et al. 2019b; Wu et al. 2020)
		distal	*OsHSP82A, OsHsfB2c*		↑	
			*OsCam1-1, LEA, OsPYL, RCAR3, OsSAPK5, OsJAZ8*		↓	
	poly(A) tail dynamic: tail lengthen		*HSP32, HSP21, HSP70, HSP70-3, HSP40, HSP101, HSP17.6II, HSP17.6C, AT1G59860, AT4G12400, AT3G09350, AT4G36040*		↑	
Cold	/		proximal	*AT1G11280, AT3G13110*	↑	(Zhang et al. 2022)
			distal	*AT5G53420*	↑

**Table 2:** Dynamic features of plant APA genes related to biotic stress responses

Biotic Stress							
Species	Stress resistance related			Phenotype changing related			Reference
	Poly(A) site usage	Gene	Expression level	Poly(A) site usage	Gene	Expression level	
Bacterium *Xanthomonas oryzae pv. Oryzae*	proximal	*OsWAK14*	↑	distal	*SGR, YL1, ASL2, OsFBK12*	↓	(Zhang et al. 2015; Ye et al. 2019b)
	distal	*OsPti1a, OsDR8, SRZ1*	↓				
		*OsSSI2*	/				
Fungus *Magnaporthe oryzae*	proximal	*OsJAZ1*	↑	*/*			(Shun et al. 2016; Ye et al. 2019b)
	distal	*OsAOC*	↓				
Virus Rice strip virus	proximal	*OsWRKY46, OsWRKY76, OsRAR1, OsRLCK107, OsDR8, DPF, POD (resistant rice)*	↑	proximal	*NYC1*	↑	*(*Sun and Jiang 2006*; *Ye et al. 2019b)
	distal	*OsNPR1, POD (susceptive rice)*	↓	distal	*YGL1*	↓	

Under stress conditions, plants often exhibit dynamic shifts in their 3' UTR lengths, with some undergoing lengthening while others experience shortening. It is important to note that key genes involved in defense mechanisms often follow distinct trajectories, deviating from the generalized lengthening or shortening trends. Tables 1 and 2 reveals a diverse array of dynamic patterns. With few exceptions, the use of most distal poly(A) sites is accompanied by less expression, and the proximal poly(A) sites are along with a higher expression level. Th intricate mechanisms that govern the relationship between poly(A) site selection and gene expression level remain elusive in plants. However, by examining the response of APA genes to various stresses, it becomes apparent that the selection of poly(A) sites and the corresponding gene expression levels play a role in bolstering plant resistance and mitigating the damage. For instance, the NADP-malic enzyme gene (*NADP-ME2*), *DCA1* and *OsRZFP34* all exhibit a trend of 3’UTR lengthening, yet they showcase either upregulation or downregulation, indicating diverse strategies to enhance stress tolerance in plants (Cui et al. 2015; Ye et al. 2019b).

As to poly(A) tail, its lengthening is generally accompanied by an increase in gene expression level (Cui et al. 2015; Ye et al. 2019b), which is contrary to another study suggesting that short poly(A) tails are highly expressed genes’ conserved feature (Lima et al. 2017). However, this discrepancy can be attributed to the outcome of stress responses. It is reasonable to assume that the stability maintained by long tails surpasses the translation efficiency provided by short tails. Nevertheless, poly(A) tails have far more complex roles beyond promoting translation and preventing degradation of mRNA. A recent research suggests that poly(A) tail together with cytoplasmic polyadenylate-binding protein (PABPC) regulate poly(A) tail removal, the rate of translation elongation, mRNA decay and other gene regulations (Passmore and Coller 2022).

Further research is necessary to elucidate the correlation between poly(A) tail and stress response in plants.

**Fig. 1:**
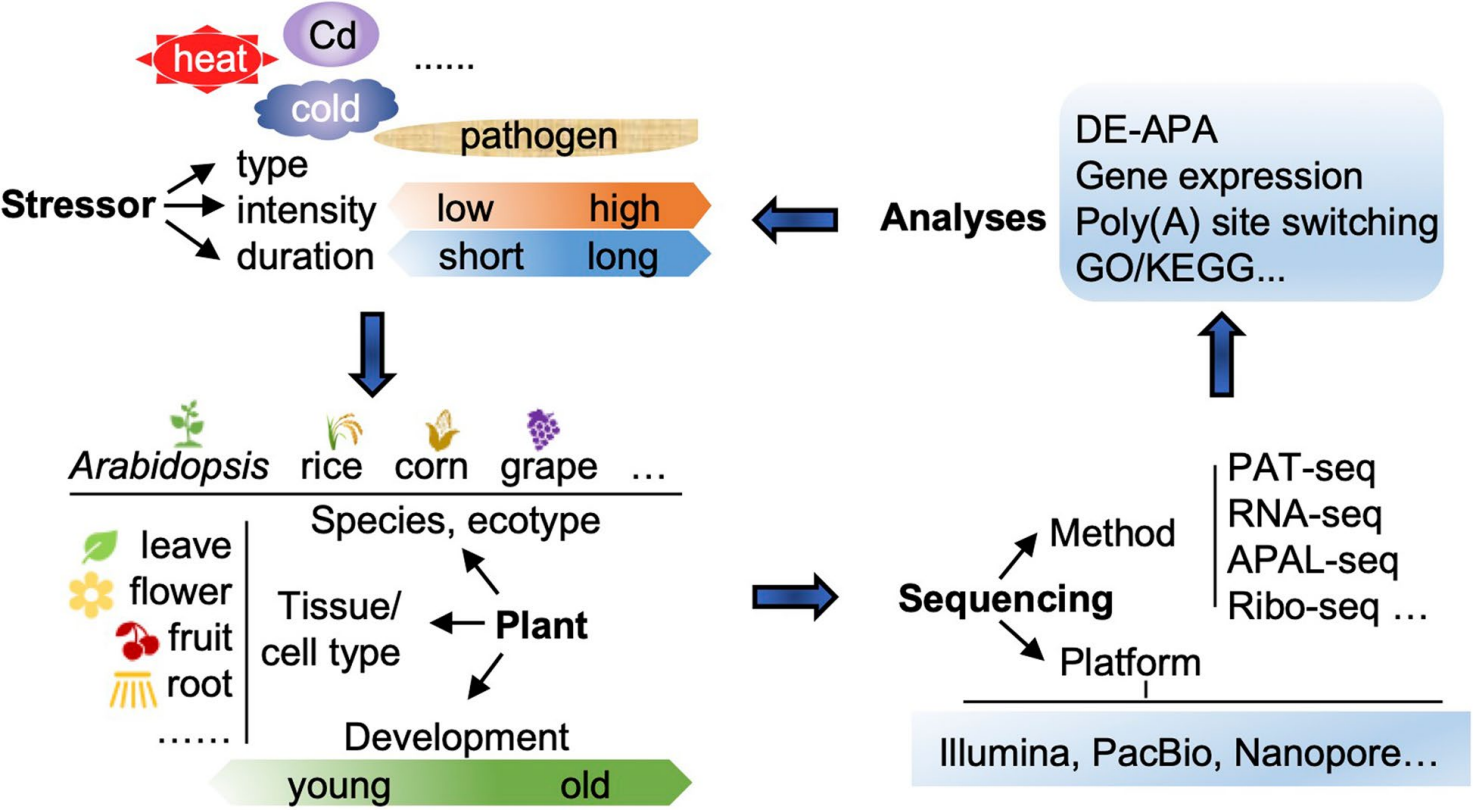
Approaches to study stress induced APA dynamics. There are several points should be considered: stress type and intensity, plant tissue and developmental stage selections, and sequencing platform selection. The type of stress and its intensity can impact APA dynamics. The selection of plant tissue is crucial as APA dynamics often exhibit tissue and spatial heterogeneity. The choice of sequencing platform depends on the research objectives, and the analysis methods are based on the sequencing method

Upon reviewing these cases, we identified some factors influencing APA dynamic related to stress responses and a research flow chart (Fig. 1). The following factors should be taken into consideration:

1. Stress type and intensity: The type of stress and its intensity can impact APA dynamics. For example, different types of heat stress (abrupt or gradual) have been shown to result in distinct APA tail length dynamics in *Arabidopsis*; similarly, the concentration and duration of Cd exposure in rice and *Arabidopsis* roots can influence poly(A) site switching (Cao et al. 2019; Ye et al. 2019b; Wu et al. 2020).
2. Plant tissue selection: The selection of plant tissue is crucial as APA dynamics often exhibit tissue and spatial heterogeneity. For instance, the APA dynamics in root hair cells, non-hair epidermal cells, and whole root tip under Cd stress (Cao et al. 2019).
3. Sequencing platform selection: The choice of sequencing platform depends on the research objectives. Deep RNA sequencing by Illumina fits PAT-seq, but PacBio would be needed for APAL-seq because Illumina platform has difficulty to sequence through homopolymers like a long tract of poly(A). While both PacBio and Nanopore have smaller through-put, they are excellent for obtaining long reads and other information like alternative splicing (PacBio) or RNA modifications (Nanopore). Ribo-seq can be used to confirm the efficacy of the outcome of APA by examine if the transcripts can be loaded to the ribosomes for translation (Erhard et al. 2018).
4. For sequencing library construction methods, the selection of tissue or cell-type level, or single cell level APA analysis, there are ample of selections based on the questions being asked. RNA-seq and its variant methods in different sequencing platforms are good to obtain full-length mRNA transcripts, but lack of precise 3’-end information, although they can be used to extract some APA information (Ye et al. 2018; Ye et al. 2023). PAT-seq (or similar methods) is designed specifically collect large amount of poly(A) sites with minimum costs (Ma et al. 2014b). An updated PAT-seq protocol termed QPAT-seq used unique molecular identifier (UMI) technology to reduce the impact of amplification for better quantitation of original transcripts (Lin et al. 2021a). The PAT-seq can also be used for quantitative analysis of polyadenylated transcript isoforms with rich details of their 3’-ends (Wu et al. 2011a). While no single cell specific poly(A) site collection protocol is available yet, single cell RNA-seq data from the 10X Genomics Chromium work follow can be used to extract single cell level poly(A) sites (Ye et al. 2020a; Wu et al. 2021). Additionally, alternative protocols based on PacBio or Nanopore platforms can also be adapted for APA site analysis (Liu et al. 2019; Long et al. 2021a).

These considerations and the proposed flowchart provide a systematic approach to studying APA dynamics in relation to stress responses in plants.

## Common mechanism of APA generation in response to stresses

**Fig. 2:**
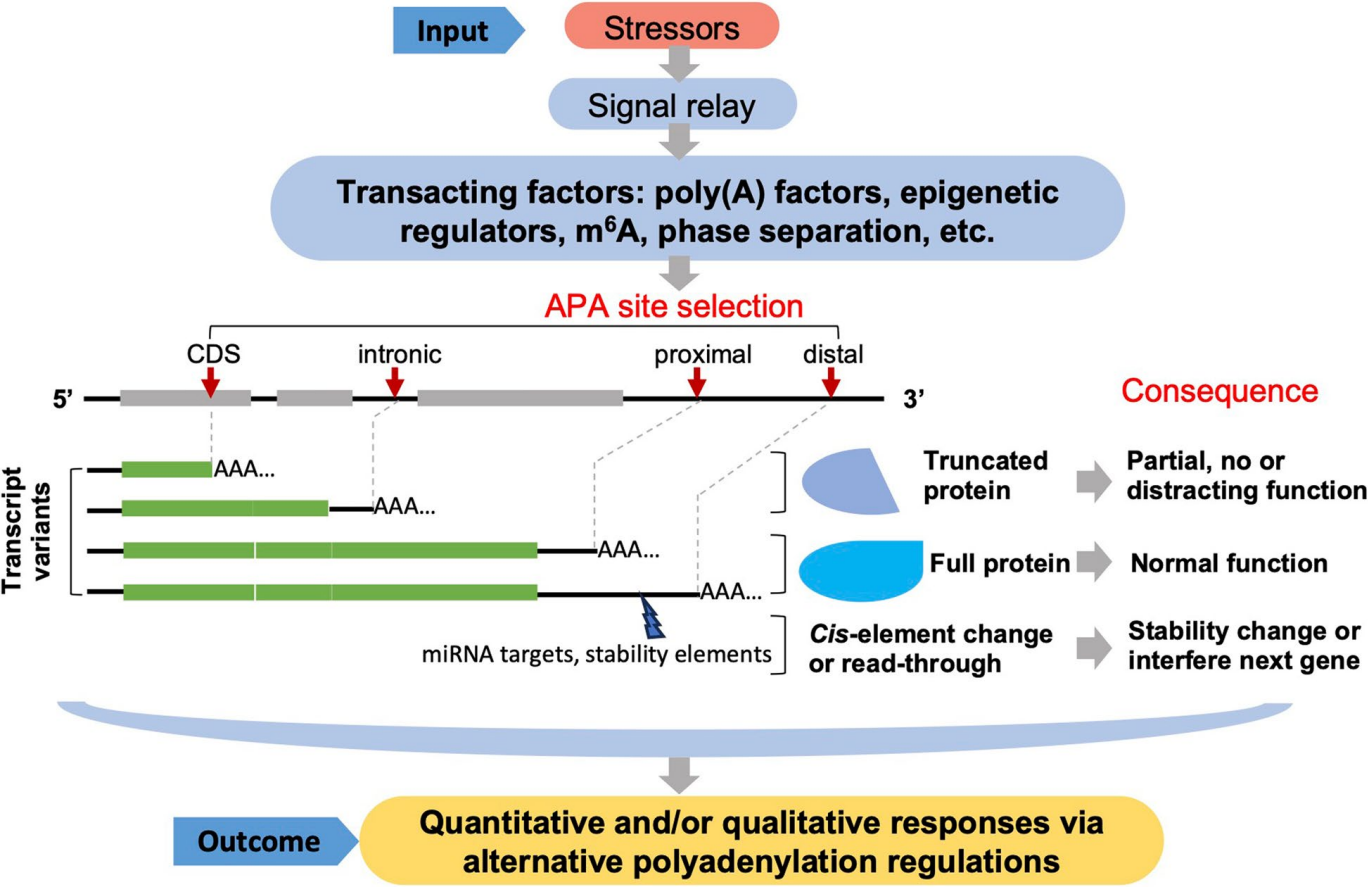
A general model for transacting factors mediated stress responses through APA dynamics. Black lines represent gene sequences, thicker grey lines represent exons, red arrows point to poly(A) sites, “AAA…” represent the poly(A) tail. Poly(A) site switching to noncanonical regions, like at intronic or protein coding sequences (CDS) region, will alter the mRNA variants and produce a smaller truncated protein different from that of the full-length mRNA product. Proximal and distal poly(A) site switching within 3’UTR would shape the 3’ end profile without altering the protein sequences, but it is highly related to mRNA stability and/or gene expression level potentially through miRNA or stability factors. In some cases, transcription by-passing the normal poly(A) site may result in “read-through” into the next gene, which could cause gene silencing effect of the next gene. The impacts of APA on transcripts and gene expression are listed on the right, but not all consequences are applicable to all transcripts. The fate of individual kind of transcripts are circumstantially influenced by different factors

Poly(A) site switching are widely found in all cases presented above, which seems to be a ubiquitous pathway in plant response to a variety of stresses. Stressors are sensed via plant sensors, and the latter transduce the signals to many TFs including poly(A) factors, epigenetic regulators, m6A factors, liquid–liquid phase separation related factors, potentially in a tissue specific and developmental stage dependent manner. These TFs play a role in selecting different play(A) sites of related genes (Fig. 2). The switching of different poly(A) sites could be classified into two types: poly(A) site switching within 3’ UTR and to noncanonical regions (not at a defined 3’UTR; Fig. 2). When a poly(A) site is switched to noncanonical regions, like intron or CDS region, it alters the mRNA isoform and produces a smaller protein (truncated protein) different from full length mRNA product. Proximal or distal poly(A) site switching within 3’UTR just shapes the 3’ end profile (the length of 3’ UTR) without altering the protein primary structure. However, different length of 3’ UTR are highly related to mRNA stability and gene expression level. As in cancer cells, lengthening 3’UTR are highly related to cellular senescence and gene express decreased (Chen et al. 2018). Of course, these two effects could happen together in response to a stress. It worth to mention that, for simplicity purposes, we did not include alternative transcription start site and alternative splicing in this model. Indeed, APA site choice could be highly involved with alternative transcription start site and alternative splicing, which can interplay with APA to finally produce variety of transcript isoforms (Li et al. 2017a; Alfonso-Gonzalez et al. 2023; Vlasenok et al. 2023).

APA related TFs, primarily poly(A) complex proteins, are potential primary regulators linking stress and APA dynamics. Among these factors, CPSF30 may play a major role of stress induced APA. *Arabidopsis CPSF30* (At1G30460), encodes a subunit of PA complex CPSF30 (30-kD, CPSF30-S) and a larger plant specific 65-kD protein (CPSF30-L) (Zhang et al. 2008). As a component of plant PA complex, CPSF30-S influences many plant APA events, and its mutant *oxt6* showed a large number of genes altering their poly(A) sites, implying the critical role of CPSF30-S in controlling APA (Thomas et al. 2012). CPSF30-S is also an important factor in oxidative signaling and cellular signaling through its interactions with calmodulin which inhibits its RNA-binding activity, and its redox sensitive function also suggest its role in calcium signaling and potential environmental stimuli sensing (Hunt et al. 2008; Chan et al. 2014). The function of APA and various signal response enable CPSF30-S connects stress response to APA processes. Plant specific protein CPSF30-L mainly connects nitrate signaling, mRNA m^6^A modification and APA: CPSF30-L possess m^6^A binding domain YT512-B homology (YTH) and recognize m^6^A-modified FUE to control poly(A) site choice (Li et al. 2017b; Hou et al. 2021; Song et al. 2021).

In all case studies described herein, ROS related genes or signals are widely identified in plants response to many kinds of stressors, especially in Cd stress, high salt, hypoxia and cold stresses. It is consistent with previous studies that ROS mediate plant signaling when they are under some severe environmental conditions (Zhang et al. 2008)*.* It’s worth to mention that CPSF30 play a vital role in ROS response as discussed earlier. Taking all together, we reckon that CPSF30 is a special universal factor to regulate stress induced APA dynamics, and its regulation is closely related to ROS response in plants.

FIP1 is another APA factor one cannot neglect. It is a component of the PA complex, interacting with poly(A) polymerase 1. FIP1-mediated poly(A) site selection contributes to salt tolerance in *Arabidopsis*, reflected in increased poly(A) site usage within the 5’ UTR and coding region (Tellez-Robledo et al. 2019). FIP1 also interact with CPSF30-L, mediating nitrate-signaling pathway especially in dividing cells in plants, with its CIPK8 and CIPK23 regulation functions (Wang et al. 2018). The role of another poly(A) factor *Arabidopsis* PCFS4 on circadian control served a case of environmental response also (Xing et al. 2013).

These findings exemplify that poly(A) factors play significant roles in stress-induced APA dynamics, connecting stress responses, signaling pathways, and gene expression regulation. Their interactions with other factors and their involvement in various physiological processes highlight their importance in plant stress responses.

## Conclusions and prospects

The dynamics of mRNA APA contribute substantially to the response mechanism when plants exposed to stress biotically and abiotically. Global APA changes and APA site switching are the two most prominent types alterations under stresses. Others like coordination of APA with other co-transcriptional events (alternative splicing, alternative transcription start sites), poly(A) tail features, and the behavior of stress induced transacting factors should not be underestimated either. Additional factors that are capable of modulating APA (by extension, to stress responses) are yet to be found. The rapidly evolving high throughput sequencing platforms and the establishment of new, more sensitive sequencing methods will also make substantial contributions to APA and stress response researches. It would be far more reaching to examine the stress induced APA in all kinds of plant materials.

However, there are still needs for deeper understanding of the pathways that are impacted by APA during stresses. Moreover, genetic and physiological confirmations of biological functions of the transcripts produced by APA remain a bigger challenge.

There are two potential directions for further investigations:

1. Specific gene investigations. Majority of the APA studies discussed herein primarily operate on the transcriptome level, and the methodologies employed for APA analysis are intricately intertwined with sequencing platforms. By following the research framework outlined in Fig. 1, a comprehensive understanding of the overarching dynamic in APA under specific environmental pressures can be garnered, further validation and more in-depth experiments are imperative. Consequently, we propose the implementation of studies centered around several specific genes. These genes are particularly poised to exert critical roles in distinct stress responses and are tightly regulated by APA. Additionally, the inclusion of key regulatory factors, such as TFs, will be integral to the investigation. The studies highlighted within this article represent a valuable repository of potential candidate genes for future explorations.
2. Single-cell level investigations. It is noteworthy that APA dynamics in response to Cd stress tend to exhibit tissue-specific patterns, particularly evident in roots, as previously discussed. This observation unveils a promising avenue for novel discoveries. Consequently, an intriguing research direction involves conducting investigations at the single-cell level. However, it is important to acknowledge that isolating distinct tissues and procuring tissue-specific materials without artificial disturbance during material preparation will present a substantial experimental challenge. Moreover, meticulous consideration must be given to the methodologies employed for constructing sequencing libraries and selecting appropriate sequencing platforms. This holistic approach is vital to ensure the accuracy and relevance of the findings in this nuanced field of study.

Overall, understanding the dynamics of APA in response to stress holds great potential for unraveling the molecular mechanisms underlying stress tolerance in plants, and it may provide valuable insights for improving crop productivity as well as resilience in a changing global environment.

## Data Availability

Not applicable.

## Abbreviations

APA: Alternative polyadenylation
AS: Alternative splicing
Cd: Cadmium
CFIm: Cleavage factor I mammalian
CFIIm: Cleavage factor II mammalian
CPSF: Cleavage and polyadenylation specificity factor
CstF: Cleavage stimulation factor
DEG: Different expression gene
EA-1: Elsinoe ampelina
FUE: Far-upstream elements
lncRNAs: Long non-coding RNAs
m^6^A: N^6^-methyladenosine
ncRNA: Non-coding RNA
NUE: Near-upstream elements
PABPC: Cytoplasmic polyadenylate-binding protein
PA complex: Polyadenylation factor complex
PACs: Poly(A) site clusters
PAL: Poly(A) tail length
PAT-seq: Poly(A) Tag sequencing
Poly(A): polyadenylation
SBPH: Small brown planthopper
R gene: Disease-resistance gene
RNA-Seq: RNA Sequencing
ROS: Reactive oxygen species
RSV: Rice strip virus
SHAPE: Selective 2’-hydroxyl acylation analyzed by primer extension
TFs: Transacting factors
TEs: Transposable elements
UTR: Untranslated region
Xoo: Xanthomonas oryzae pv. Oryzae
YTH: YT512-B homology domain
